# Real-World Evidence of the Impact of the COVID-19 Pandemic on Lung Cancer Survival: Canadian Perspective

**DOI:** 10.3390/curroncol31030119

**Published:** 2024-03-19

**Authors:** Jason Agulnik, Goulnar Kasymjanova, Carmela Pepe, Jennifer Friedmann, David Small, Lama Sakr, Hangjun Wang, Alan Spatz, Khalil Sultanem, Victor Cohen

**Affiliations:** 1 Department of Internal Medicine, Division of Pulmonary Diseases, Jewish General Hospital, McGill University, Montreal, QC H3T 1E2, Canada; jason.agulnik.med@ssss.gouv.qc.ca (J.A.); carmela.pepe@mcgill.ca (C.P.); david.small@mcgill.ca (D.S.); lama.sakr@mcgill.ca (L.S.); 2 Department of Medical Oncology, Jewish General Hospital, McGill University, Montreal, QC H3T 1E2, Canada; jennifer.friedmann.med@ssss.gouv.qc.ca (J.F.); victor.cohen@mcgill.ca (V.C.); 3 Department of Pathology, Jewish General Hospital, McGill University, Montreal, QC H3T 1E2, Canada; hangjun.wang@mcgill.ca (H.W.); alan.spatz@mcgill.ca (A.S.); 4 Department of Radiation Oncology, Jewish General Hospital, McGill University, Montreal, QC H3T 1E2, Canada; khalil.sultanem@mcgill.ca

**Keywords:** lung cancer, COVID-19, survival

## Abstract

**Background:** The effect of COVID-19 on treatment outcomes in the literature remains limited and is mostly reported either as predictive survival using prioritization and modeling techniques. We aimed to quantify the effect of COVID-19 on lung cancer survival using real-world data collected at the Jewish General Hospital, Montreal. **Methods:** This is a retrospective chart review study of patients diagnosed between March 2019 and March 2022. We compared three cohorts: pre-COVID-19, and 1st and 2nd year of the pandemic. **Results:** 417 patients were diagnosed and treated with lung cancer at our centre: 130 in 2019, 103 in 2020 and 184 in 2021. Although the proportion of advanced/metastatic-stage lung cancer remained the same, there was a significant increase in the late-stage presentation during the pandemic. The proportion of M1c (multiple extrathoracic sites) cases in 2020 and 2021 was 57% and 51%, respectively, compared to 31% in 2019 (*p* < 0.05). Median survival for early stages of lung cancer was similar in the three cohorts. However, patients diagnosed in the M1c stage had a significantly increased risk of death. The 6-month mortality rate was 53% in 2021 compared to 47% in 2020 and 29% in 2019 (*p* = 0.004). The median survival in this subgroup of patients decreased significantly from 13 months in 2019 to 6 months in 2020 and 5 months in 2021 (*p* < 0.001). **Conclusions:** This study is, to our knowledge, the largest single-institution study in Canada looking at lung cancer survival during the COVID-19 pandemic. Our study looks at overall survival in the advanced/metastatic setting of NSCLC during the COVID-19 pandemic. We have previously reported on treatment pattern changes and increased wait times for NSCLC patients during the pandemic. In this study, we report that the advanced/metastatic subgroup had both an increase in the 6-month mortality rate and worsening overall survival during this same time period. Although there was no statistical difference in the proportion of patients with advanced disease, there was a concerning trend of increased M1c disease in cohorts 2 and 3. The higher M1c disease during the COVID-19 pandemic (cohorts 2 and 3) likely played a crucial role in increasing the 6-month mortality rate and leading to a reduced overall survival of lung cancer patients during the pandemic. These findings are more likely to be better identified with longer follow-up.

## 1. Introduction

The World Health Organization (WHO) declared COVID-19 a pandemic in March 2020. The COVID-19 pandemic has not only posed unprecedented challenges to healthcare systems worldwide but has also significantly impacted the landscape of cancer care, particularly for individuals battling lung cancer. Lung cancer is one of the most prevalent and deadly forms of cancer globally. It requires timely and comprehensive treatment interventions to optimize patient outcomes. The initial lockdown measures imposed by the government-recommended COVID-19 protocol in many countries led to widespread disruptions in routine medical services, including cancer screenings, diagnostic procedures, and elective surgeries. Fear of contracting the virus also deterred patients from seeking medical care for non-COVID-19-related conditions, including lung cancer. Numerous studies have shed light on a concerning phenomenon within the realm of oncology: a marked decline in the detection of new lung cancer cases coupled with a concerning shift in the stage at which these malignancies are being diagnosed. These studies collectively underscore the profound impact that the pandemic, particularly its initial lockdown measures, has had on lung cancer diagnosis and presentation [1,2,3,4,5,6,7,8,9,10,11].

Our recent publications have demonstrated the immense impact of the COVID-19 pandemic on lung cancer care. In the first year of the pandemic, disruptions in the health care systems resulted in a significant decline in new lung cancer diagnoses. However, as the pandemic persisted into its second year, an alarming shift in the trajectory of new lung cancer diagnoses emerged. Contrary to the earlier decline observed, we noticed a significant resurgence in new lung cancer cases, surpassing even pre-pandemic levels. We also observed a significant rise in cases having multiple extrathoracic metastases. The reason behind this we believe is the disruption of health care services, prolonged wait times for diagnosis and treatment, and the slow return to regular healthcare services after the pandemic’s initial impact [12,13].

Until this point, data on the effect of lung cancer treatment outcomes during the COVID-19 pandemic have been scant and the limited existing studies have reported on expected or short-term survival rates using prioritization and modeling techniques. Various models have been proposed to estimate expected mortality from simple-count to time series-type analyses. More recently, analytical models providing more comprehensive mortality assessments using machine-based algorithms have been introduced. Predictions of the impact of the COVID-19 pandemic on lung cancer deaths have varied across models, with differences, at least in part, due to differing assumptions regarding the extent of disruptions to cancer care in different settings. The scarcity of data regarding treatment outcomes has prompted us to look deeper into own experiences with lung cancer treatment and its outcomes during the pandemic. This study is based on real-world data and experiences. Our goal is to show how the COVID-19 pandemic has affected overall survival and mortality among lung cancer patients.

## 2. Materials and Methods

### 2.1. Population

As previously described in our 2 other published studies, this study included patients who were diagnosed with lung cancer between March 2019 and March 2022 and received treatment and follow-up care at the Peter Brojde lung Cancer Center, Jewish General Hospital (JGH) [12,13]. JGH is a McGill University-affiliated hospital that offers a range of services related to the diagnosis, treatment, and management of cancer, such as diagnostics, treatment, supportive care, clinical trials, counseling, education and support programs, and follow-up care.

Study protocol # 2021-2655 was approved by the CIUSSS West-Central Montreal Institutional Review Board on 22 January 2021.

Patients were divided into three cohorts:

- Cohort #1 (2019): Pre-COVID-19
- Cohort #2 (2020): 1st year of COVID-19
- Cohort #3 (2021): 2nd year of COVID-19

### 2.2. Objectives

The primary aim of this study was to evaluate how the COVID-19 pandemic influenced mortality and overall survival rates in lung cancer patients.

### 2.3. Statistical Analysis

In the aim of comprehensive data acquisition, we reviewed electronic medical charts to extract patient information, including demographics, disease characteristics and outcome metrics.

In this study, we summarized continuous variables by calculating means, standard deviations, medians and ranges. Categorical variables were summarized using frequency distributions and percentages. We determined the significance of differences using chi-square statistics. We assessed overall survival, which is the time from diagnosis to death, or the last follow-up using the Kaplan–Meier method (Log Rank test). Mortality was the count of patients who died in a given time in the study population. Short-term 6-month mortality was calculated using the live table. We conducted survival Cox regression analysis to identify potential prognostic factors incorporating variables such as age, sex, ECOG PS, stage, and year of diagnosis. All statistical analyses were performed using the SPSS software, version 24.0 (SPSS, Chicago, IL, USA). We conducted two-tailed tests, considering *p*-values of 0.05 or lower as significant. The dataset was locked on 30 November 2022.

## 3. Results

### 3.1. Patient Characteristics

Over the course of 3 years, our clinic provided care to 500 newly diagnosed lung cancer patients (Table 1). After excluding second opinions (patients seeking an additional opinion to gain more informed choices regarding their treatment options), our study encompassed 417 patients. As previously reported, the analysis revealed a 21% drop in new diagnoses in Cohort 2 compared to Cohort 1 and a significant increase of 79% in Cohort 3 compared to Cohort 2 (*p* = 0.004).

We present patient characteristics in Table 2. Most of the baseline characteristics were similar between the three Cohorts except for smoking status and sex. In Cohort 3, more lung cancer patients were female (52%) and current/former smokers (85%) at the time of diagnosis (*p* = 0.054) compared to Cohorts 1 and 2.

As shown in Table 2, the overall proportion of advanced/metastatic stage lung cancer remained relatively consistent during the pandemic when compared to the year before. However, we did observe a significant increase in the numbers of M1c cases (multiple extrathoracic sites) in Cohort 2 and 3, with a percentage of 57% and 51%, respectively, as opposed to Cohort 1, which had a lower rate of 31% (see Table 3). In addition, there was a decrease in M1b (single extrathoracic metastasis) cases in Cohorts 2 and 3.

### 3.2. Front-Line Treatment

The front-line treatments received are outlined in Table 4. Overall, 345 (83%) patients received a first definitive treatment (FDT) which comprised either surgery, radiosurgery, or systemic treatment. The percentage of patients receiving front-line definitive treatment was consistent across all three cohorts: 85%, 82% and 84%, respectively.

During the initial year of the pandemic (Cohort 2), a significant shift in treatment paradigms was observed, marked by a surge in the utilization of radiosurgery as the primary modality of intervention in early-stage cases (Table 4). This rise in radiosurgical interventions were observed with a significant decline in the frequency of surgical procedures within the same cohort, highlighting a divergence in therapeutic approaches during the COVID-19 era. However, during the 2nd year of the pandemic (Cohort 3), a remarkable normalization in treatment patterns was observed. Both the utilization of radiosurgery and the frequency of thoracic surgical interventions returned to pre-COVID-19 levels. Interestingly, systemic chemotherapy and targeted therapy regimens remained largely unaffected by the pandemic-induced measures, with no statistically significant alterations observed (*p* > 0.05).

### 3.3. Lung Cancer Mortality and Survival

Table 5 provides a comprehensive overview of patients who died within the 6 months following their initial diagnosis. Our analysis revealed a consistent pattern across all three cohorts, manifesting no difference in the observed 6-month mortality rate among patients diagnosed at early and locoregional stages. Nonetheless, while not reaching statistical significance, there existed a marginal elevation in 6-month mortality among early and locoregional stages patients within Cohort 2 and Cohort 3 during the initial year of the pandemic, as compared with the pre-pandemic era (Table 5). Notably, within the second year of the COVID-19 pandemic (Cohort 3), the mortality rate among these patients reverted to pre-pandemic levels.

However, a concerning trend emerged among patients with advanced or metastatic lung cancer. In Cohort 1, representing pre-pandemic data, the mortality rate at 6 months was at 29% in this subgroup. However, as we progressed through the pandemic, a distressing escalation in mortality rates became more evident. The 6-month mortality rate was 47% in Cohort 2 and continued to increase to 53% in Cohort 3, with a *p*-value of 0.004.

**Figure 1:**
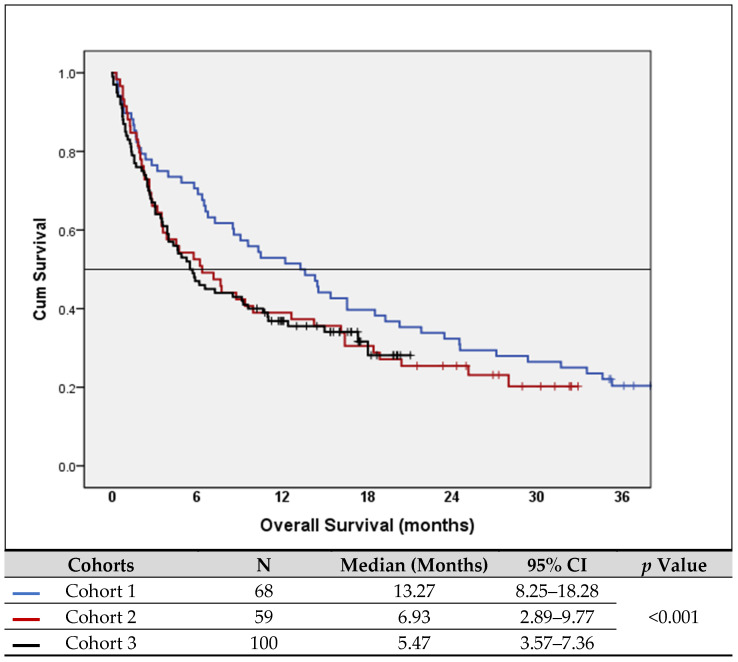
Survival of patients with advanced/metastatic disease.

The overall median survival of all 417 patients included in this study showed a trend toward better survival in patients diagnosed in Cohort 1 (the year before the pandemic) but this difference was not statistically significant. However, in the subgroup of patients with advanced/metastatic-stage lung cancer, Cohort 1 had a median survival of 13.27 months, while those in Cohort 2 and Cohort 3 had statistically significant shorter survival times (6.93 and 5.47 months, respectively), with a *p* value < 0.001 (Figure 1).

In the analyses of prognostic factors, two Cox models were used. The first model, including all 417 patients, found that non-smokers, those with an ECOG PS of 0–1, younger individuals and those with early-stage disease tended to have significantly better survival; however, sex and the year of diagnosis did not impact survival. The second model focused on patients with advanced/metastatic-stage cancer. Older age (*p* < 0.001), poor PS (*p* < 0.001), positive smoking history (*p* < 0.001) and diagnosis during COVID (Cohorts 2 and 3) (*p* = 0.042) were found to be significantly associated with poor survival (please see Figure 1 and Table 6).

## 4. Discussion

In our previous research publications [12,13], we highlighted a significant decline in the incidence of new lung cancer cases during the initial phase. This observation is consistent with widespread global reports documenting similar trends [1,2,3,4,5,6,7,8,9,10,11]. Following the decline witnessed initially, we observed a significant resurgence in the incidence of new cases, with an escalation of 79% during the second year of the pandemic. There were also some changes noticed in the demographics of the patients. In our study, we observed a 10% increase (with a *p* value of 0.054) in smoking prevalence among lung cancer patients. This finding resonates with the observation made by the Editorial Board of Lancet Respiratory Medicine. They linked this increase to various factors such as the implementation of lockdown measures, heightened stress levels and the effects of social isolation. [14]. There is ongoing multicenter Italian study on the role of tobacco smoking on the severity and progression of COVID-19 in hospitalized patients [15]. Additionally, our analyses revealed a modest but noticeable uptrend in the proportion of female patients from 44% in 2019 to 52% in 2021. This shift in gender distribution aligns with the study conducted by Mangole et al., where they highlighted significant gender disparities in lung cancer diagnoses during the pandemic’s initial phase compared to the pre-pandemic period. Their findings indicated a 17.6% decrease in diagnoses among males contrasted with a 14.1% increase among females [16]. These gender-specific variations emphasize the significant impact of COVID-19 on cancer epidemiology and stress the importance of considering gender dynamics in healthcare research and policy formulation.

While much discussion has centered on the delays in diagnosis and treatment following the COVID-19 pandemic, few studies have explored how these delays impact the stage at which cancer is diagnosed. Although our study did not demonstrate a shift in lung cancer staging, we did observe an increase in proportion of symptomatic cases during the pandemic, with a significant rise in cases having multiple extrathoracic metastases (M1c). The numbers of patients with multiple extrathoracic metastases increased by 20% in 2020 and 2021 compared to the pre-COVID era. This trend was in line with findings by Flores et al., who suggested that the rise in late-stage diagnoses during pandemic surges may be attributed to the fact that only individuals experiencing symptoms or acute medical events necessitating immediate attention sought hospital care [2]. Similarly, Orelaru et al. identified more cases of stage IV NSCLC during COVID-19 compared with early-stage NSCLC (stages 1A and 3A) in the pre-COVID-19 era [17]. These data underscore the need of early detection efforts, where interventions may offer the greatest potential for therapeutic efficacy and improved patient outcomes.

The significance of mortality and survival as pivotal endpoints in lung cancer research and clinical practice cannot be overstated, with initial staging serving as one of the most important prognostic factors. Short-term mortality and survival rates for early- or locoregional-stage lung cancer typically exhibit a more favorable outlook compared to advanced stages, due to the advantages of early detection enabling timely intervention and potentially curative treatment modalities such as surgery, radiation therapy, and targeted therapies. In our investigation, spanning a 2-year period of the COVID-19 pandemic, we did not observe any significant difference in outcomes when compared to pre-pandemic levels. However, it is worth noting that while statistical significance was not reached, there was a slight elevation in 6-month mortality among early- and locoregional-stage patients within Cohort 2 and Cohort 3 during the first year of the pandemic, compared to the pre-pandemic year. We postulate that this might be related to COVID-19 infection outcomes in lung cancer patients. In support of our finding, Garassino et al. reported a high mortality rate (33%) among lung cancer patients infected with COVID-19 [18]. Our previous work demonstrated a shift in the utilization of surgery and radiation therapy, and we hypothesize that there may be a decrease in survival. However, no significant variance in survival was detected, which we attribute to the short duration of follow-up and the relatively small number of early-stage patients. This underscores the need for further investigation and longer-term follow-up to evaluate the full impact of the pandemic on early-stage lung cancer outcomes.

Unfortunately, the diagnosis of later-stage lung cancer cases led to markedly inferior survival evidenced by near-doubling of the 6-month mortality rates in 2021 when compared to pre-COVID era (*p* = 0.004). Furthermore, the median survival duration within this subgroup demonstrated a substantial decrease from 13 months in 2019 to 5 months in 2021 (*p* < 0.001). It is important to note that these findings are based on relatively short follow-up period, and it is expected that long-term follow-up may reveal an even more pronounced decline in survival rate over time. These findings serve as a reminder of the profound impact of the COVID-19 pandemic on the trajectory of lung cancer outcomes, particularly for those presenting in the most advanced stages of the disease.

In our comprehensive multivariate analysis, we revealed the following: among the established prognostic factors such as advanced age, compromised ECOG performance status, and a history of smoking, there emerged a noteworthy finding. Specifically, the timing of lung cancer diagnosis during the COVID-19 pandemic emerged as an important variable significantly linked to mortality, particularly within the subgroup of patients with advanced- or metastatic-stage disease. This underscores an important reality: the year of diagnosis of the lung cancer during the pandemic exerts a profound influence on patient outcomes. Patients with advanced lung cancer diagnosed during the pandemic were found to have worsened overall survival. Our previous findings indicated that pandemic-related lockdown measures caused extended delays in cancer diagnosis and treatment and subsequently a shift toward a late stage of disease at presentation. This could have been a contributing factor to the poor outcomes. Additionally, we postulate that the morbidity and mortality associated with COVID-19 infection may have further exacerbated these adverse outcomes in lung cancer patients.

Our study is distinctive in providing a real-world perspective on the impact of COVID-19 on lung cancer survival in Canada, supported by real-time healthcare data. It is among the early reports, as survival data are still evolving and many publications have focused on predicted or estimated survival [14,19,20,21]. In accordance with our findings, a retrospective study from Spain presented at WCLC indicated a median survival of 6.7 months for lung cancer patients during COVID-19, with a 30-day mortality increasing from 25% before to 49% during the COVID-19 pandemic [22].

## 5. Limitations

We acknowledge that our work has potential limitations as a retrospective study. As with any single-center retrospective study, external validity is threatened, and the results may not be generalizable to other institutions. It would be very interesting to see if similar outcomes on cancer mortality from COVID-19 are observed in other centers in Canada, particularly in some of the other larger provinces and in other countries. Another important issue relates to data maturity and the short follow-up period for the early and locoregional stage of lung cancer patients. A longer follow-up time will decrease the risk of error when projecting long-term survival.

## 6. Conclusions

This study is, to our knowledge, the largest single-institution study in Canada looking at lung cancer survival during the COVID-19 pandemic. Our study looked at overall survival in the advanced/metastatic setting of NSCLC during the COVID-19 pandemic. We had previously reported on treatment pattern changes and increased wait times for NSCLC patients during the COVID-19 pandemic. In this study, we reported that the advanced/metastatic subgroup had both an increase in the 6-month mortality rate and worsening overall survival during this same time period. Although there was no statistical difference in the proportion of patients with advanced disease, there was a concerning trend of increased M1c disease in Cohorts 2 and 3. The higher M1c disease during the COVID-19 pandemic (Cohorts 2 and 3) likely played a crucial role in increasing the 6-month mortality rate and led to a reduced overall survival of lung cancer patients during the pandemic. These findings are more likely to be better identified with longer follow-up.

## Figures and Tables

**Table 1:** Number of study participants.

Study Cohorts	Included in the Study	Second Opinion (Excluded)	Total	*p* Value
Cohort 1 (2019)	130	40	170	0.004
Cohort 2 (2020)	103	8	111	
Cohort 3 (2021)	184	35	219	
Total	417	83	500	

**Table 2:** Patients’ characteristics.

Characteristics		2019 *n* = 130	2020 *n* = 103	2021 *n* = 184	
Age (mean; range)		70 (40–96)	71 (42–92)	71 (41–92)	0.991
Sex *n* (%)	Male	73 (56)	56 (54)	88 (48)	0.145
	Female	57 (44)	47 (46)	96 (52)	
Smoking *n* (%)	Former/current	99 (76)	74 (74)	156 (85)	0.054
	Non-smoker	31 (24)	29 (26)	28 (15)	
ECOG PS *n* (%)	0–1	106 (82)	84 (82)	148 (80)	0.960
	>1	24 (18)	19 (18)	36 (20)	
Stage	Early ^1^	42 (32)	33(32)	55 (30)	0.787
	Locoregional ^2^	20 (15)	11 (11)	29 (16)	
	Advanced/Metastatic ^3^	68 (52)	59 (57)	100 (54)	

^1^ T_1–3_N_0–1_M0; ^2^ T_1–4_N_2–3_M0; ^3^ T_any_N_any_M_1_ (8th edition of the TNM Classification for Lung Cancer).

**Table 3:** Number of distant metastases.

M Status	Cohort 1 *n* = 68	Cohort 2 *n* = 59	Cohort 3 *n* = 100
M1a ^1^ *n* (%)	20 (29)	14 (24)	31 (31)
M1b ^2^ *n* (%)	27 (40)	11 (19)	18 (18)
M1c ^3^ *n* (%)	21 (31)	34 (57)	51 (51)
*The two-tailed p value (2020* vs. *2019)*	0.002		
*The two-tailed p value (2021* vs. *2019)*	0.013		

^1^ Pericardial/pleural effusion or contralateral lung; ^2^ Single extrathoracic site; ^3^ Multiple extrathoracic sites.

**Table 4:** Type of first definitive treatment (FTD).

Type of FTD		Cohort 1 *n* = 110	Cohort 2 *n* = 84	Cohort 3 *n* = 151	*p*-Value
Surgery (*n*/%)		42 (38)	24 (29)	54 (36)	0.01
Radiosurgery (*n*/%)		8 (7)	20 (24)	12 (8)	0.009
CTX ^2^ (*n*/%)	Total	60 (54)	40 (47)	85 (56)	>0.05
	SOC ^1^	20 (33)	12 (30)	25 (29)	
	IO ± CTX ^3^	23 (38)	16 (40)	37 (44)	
	Targeted CTX	17 (29)	12 (30)	23 (27)	

^1^ Standard of care chemotherapy; ^2^ Chemotherapy; ^3^ Immunotherapy +/− chemotherapy.

**Table 5:** Six-month mortality rate before and during COVID-19.

Stage	Cohort	*n*	Died at 6 Months *n* (%)	*p* Value
Early stage ^1^	1 (2019)	42	1 (2)	
	2 (2020)	33	3 (9)	>0.05
	3 (2021)	55	0 (0)	
Locoregional ^2^	1 (2019)	20	3 (15)	0.407
	2 (2020)	11	3 (27)	
	3 (2021)	29	4 (14)	
Advanced/metastatic stage ^3^	1 (2019)	68	20 (29)	0.004
	2 (2020)	59	28 (47)	
	3 (2021)	100	53 (53)	

^1^ T_1–3_N_0–1_M0; ^2^ T_1–4_N_2–3_M0 +Limited SCLC; ^3^ T_any_N_any_M_1+_Extencive SCLC.

**Table 6:** Cox models of prognostic factors for survival.

**Model #1 Description**	**Variables Included in the Model**	**Reference**	**Exp(B)**	**95% CI**	***p* Value**
**All subjects**	Year of diagnosis	2019	1.098	0.926–1.302	0.284
	Age	≤65	1.024	1.021–1.269	0.005
Events = 199	Sex	Female	1.106	0.824–1.485	0.502
Censored = 218	ECOG PS	0–1	2.360	1.721–3.237	<0.001
Missing = 0	Smoking	Smoker	0.729	0.598–0.897	0.002
Total = 417	Stage	1–3A	3.438	2.663–4.438	<0.001
**Model #2 Description**	**Variables Included in the Model**		**Exp(B)**	**95% CI**	***p*** **Value**
**Advanced/metastatic stage**	Year of diagnosis	2019	1.156	1.021–1.393	0.042
	Age	≤65	1.035	1.018–1053	<0.001
Events = 167	Sex	Female	1.108	0.803–1.530	0.532
Censored = 60	ECOG PS	0–1	2.483	1.780–3.463	<0.001
Total = 227	Smoking	Smoker	0.723	0.584–0.894	<0.001

## Data Availability

The data presented in this study are available on request from the corresponding author.
